# Supplementary material for: Chromosome-Level Genome Assembly and Circadian Gene Repertoire of the Patagonia Blennie Eleginops maclovinus—The Closest Ancestral Proxy of Antarctic Cryonotothenioids
Source: Genes (Basel). 2023 May 30;14(6):1196. doi: 10.3390/genes14061196 (PMC10298351; doi:10.3390/genes14061196)
Supplement: Supplementary file 1 [file genes-14-01196-s001.zip › Fig.S2_cry3a_maps.pdf]

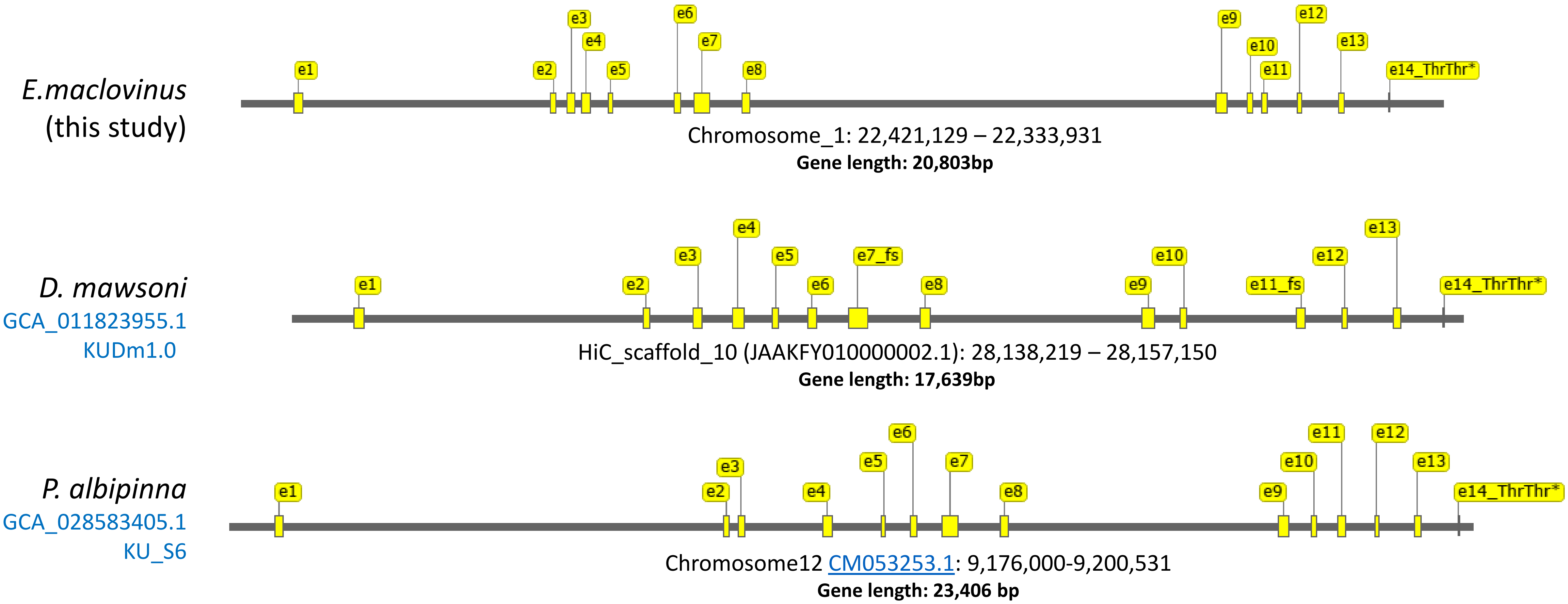

Figure S2. Manually curated and annotated *cry3a* from *D. mawsoni* and *P. albipinna* genome assembly. *E. maclovinus cry3a* gene structure is given as reference. “e” stands for exon. The two Thr residues encoded by the mini-e14 is indicated. Relative lengths of exons (rectangular boxes) and between exons are to scale.
