# Supplementary material for: Chromosome-Level Genome Assembly and Circadian Gene Repertoire of the Patagonia Blennie Eleginops maclovinus—The Closest Ancestral Proxy of Antarctic Cryonotothenioids
Source: Genes (Basel). 2023 May 30;14(6):1196. doi: 10.3390/genes14061196 (PMC10298351; doi:10.3390/genes14061196)
Supplement: Supplementary file 1 [file genes-14-01196-s001.zip › Fig.S3_Palb_arntl1.pdf]

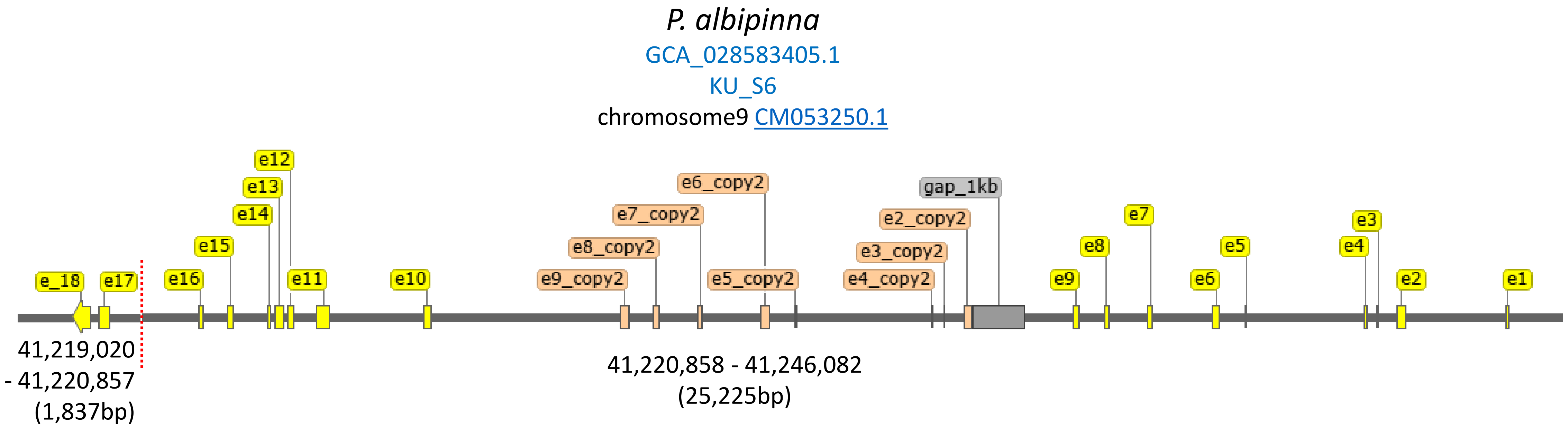

Figure S3. Errors in automated annotation of the circadian gene *arntl1a* in *P. albipinna* genome assembly. The gene is oriented in the reverse direction. The 25,225 bp to the right the red dashed line is the gene model in NCBI. Manual inspection found a repeated section of sequence upstream of a 1kbp gap spanning a second copy of exons 2 through 9, resulting in an 806 aa protein that is over 150 aa longer than typical arntl1a. This repeated segment likely resulted from genome assembly error. Also, the gene model is truncated , ending at an atypical stop codon at exon16. Manual annotation of adjacent sequence (left of the red dashed line) recovered the last two coding exons. The complete 18-exon gene (excluding the repeated e2-e9) is structurally intact, and yields a translated protein of 638 amino acids.
