# Supplementary material for: Chromosome-Level Genome Assembly and Circadian Gene Repertoire of the Patagonia Blennie Eleginops maclovinus—The Closest Ancestral Proxy of Antarctic Cryonotothenioids
Source: Genes (Basel). 2023 May 30;14(6):1196. doi: 10.3390/genes14061196 (PMC10298351; doi:10.3390/genes14061196)
Supplement: Supplementary file 1 [file genes-14-01196-s001.zip › Fig.S1_Emac_Xmac_WG_synteny.pdf]

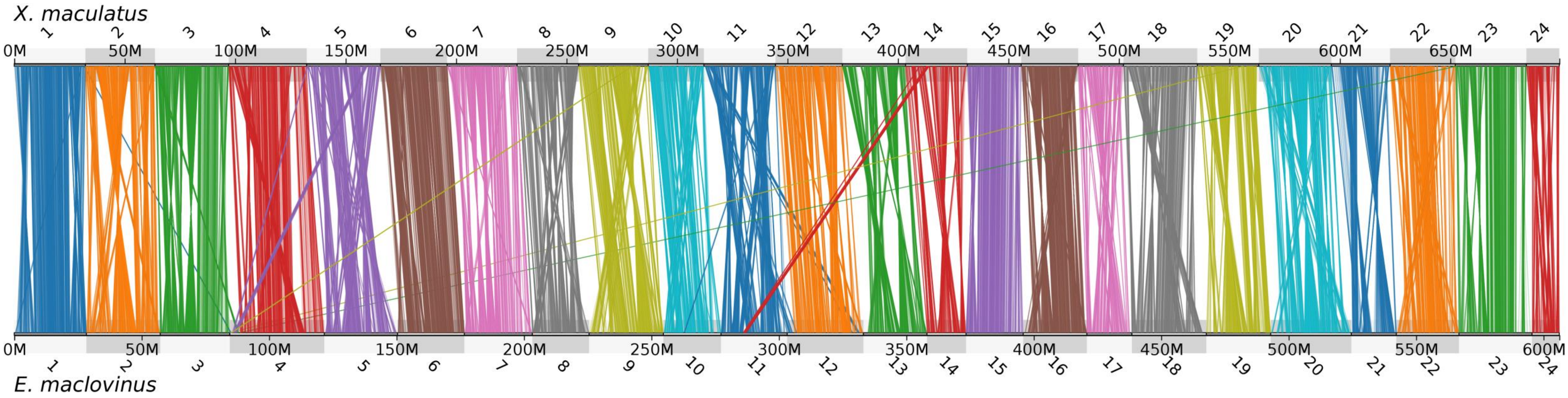

Fig. S1: Conserved genome-wide synteny between *E. maclovinus* and the platyfish, *X. maculatus*. Genome-wide conserved synteny plot displaying orthology between the 24 chromosomes of *X. maculatus* (top) and *E. maclovinus* (bottom). Each ortholog gene pair between the two species is represented by a line color-coded according to their chromosome of origin. While synteny highlights multiple intra-chromosomal rearrangements, chromosomes largely display one-to-one correspondence between the two species. In turn, the *E. maclovinus* chromosomal scaffolds were named according to their orthologous *X. maculatus* chromosome.
